# Properties and Processing of Aviation Exhaust Aerosol at Cruise Altitude Observed from the IAGOS-CARIBIC Flying Laboratory

**DOI:** 10.1021/acs.est.3c09728

**Published:** 2024-04-08

**Authors:** Christoph Mahnke, Rita Gomes, Ulrich Bundke, Marcel Berg, Helmut Ziereis, Monica Sharma, Mattia Righi, Johannes Hendricks, Andreas Zahn, Andreas Wahner, Andreas Petzold

**Affiliations:** † Forschungszentrum Jülich GmbH (FZJ), Institute of Energy and Climate Research: Troposphere (IEK-8), Jülich 52428, Germany; ‡ Deutsches Zentrum für Luft- und Raumfahrt (DLR), Institut für Physik der Atmosphäre, Oberpfaffenhofen 82234, Germany; § Faculty of Aerospace Engineering, Delft University of Technology, Delft 2600 AA, The Netherlands; ∥ Karlsruhe Institute of Technology (KIT), Institute of Meteorology and Climate Research, Karlsruhe 76131, Germany; ⊥ Institute for Atmospheric and Environmental Research, University of Wuppertal, Wuppertal 42119, Germany

**Keywords:** aviation aerosol, aerosol particle, aircraft exhaust, aircraft plume, particle emission index, aviation soot, plume detection, IAGOS-CARIBIC

## Abstract

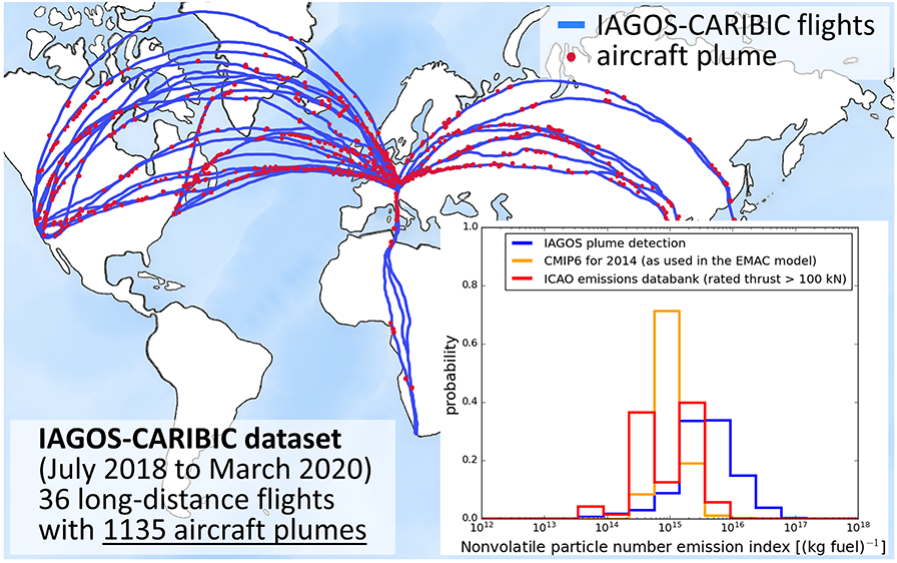

The characteristics of aviation-induced aerosol, its processing, and effects on cirrus clouds and climate are still associated with large uncertainties. Properties of aviation-induced aerosol, however, are crucially needed for the assessment of aviation’s climate impacts today and in the future. We identified more than 1100 aircraft plume encounters during passenger aircraft flights of the IAGOS-CARIBIC Flying Laboratory from July 2018 to March 2020. The aerosol properties inside aircraft plumes were similar, independent of the altitude (i.e., upper troposphere, tropopause region, and lowermost stratosphere). The exhaust aerosol was found to be mostly externally mixed compared to the internally mixed background aerosol, even at a plume age of 1 to 3 h. No enhancement of accumulation mode particles (diameter >250 nm) could be detected inside the aircraft plumes. Particle number emission indices (EIs) deduced from the observations in aged plumes are in the same range as values reported from engine certifications. This finding, together with the observed external mixing state inside the plumes, indicates that the aviation exhaust aerosol almost remains in its emission state during plume expansion. It also reveals that the particle number EIs used in global models are within the range of the EIs measured in aged plumes.

## Abstract 2

Only few measurements of aircraft exhaust aerosol are available for cruise altitude. This study utilizes IAGOS-CARIBIC data from passenger aircraft flights to analyze the aerosol properties of over 1100 exhaust plumes and puts them into context with the data available for global climate models.

## 1 Introduction

Global civil aviation is a significant contributor to anthropogenic climate change through a complex series of processes that are primarily driven by emissions from aircraft gas turbines. These emissions include carbon dioxide (CO_2_), reactive nitrogen oxides (NO_*x*_), water vapor, carbonaceous and sulfate aerosols, and a mixture of gaseous hydrocarbon (HC) compounds. Lee et al.^1^ provides an overview of the current understanding of the impact of civil aviation on climate change. The present study focuses on particulate matter emission and atmospheric processing, as their ability to form ice in contrails has a significant impact on the climate effects of aviation,^2,3^ while some studies also argued for a potential impact on natural clouds.^4−7^

Aircraft gas turbine engines release ultrafine particles with diameters smaller than 100 nm, which are generated during the combustion of hydrocarbon fuels. The emitted aerosol contains primary carbonaceous particles, including black carbon (BC) and organic carbon (OC), commonly known as soot. These particles are formed during incomplete combustion. Additionally, the aerosol contains condensation particles that nucleate and condense in the cooling exhaust gas from gaseous precursors, such as sulfuric acid and hydrocarbon vapors (for an overview, see Petzold et al.^8^). The emitted aerosol particles are classified into two groups based on their thermal stability: nonvolatile (which remains stable at temperatures above 250 °C) and volatile (which evaporates below 250 °C). For certification and regulation purposes of aircraft engines, this temperature threshold is currently set at 350 °C.^9,10^ The nonvolatile mode refers to the carbonaceous particles generated from fuel combustion, while the volatile mode includes droplets composed of sulfur and organic compounds.

According to Petzold et al.,^11^ the fraction of primary carbonaceous particles can be identified by a particle mode with a median diameter of 25 nm, as well as a weaker mode at 150 nm. The number of nonvolatile particles released per unit mass of fuel burned is heavily influenced by the type of fuel and aircraft engine technology used and has decreased significantly from the 1960s (2 × 10^15^ particles per kg fuel) to the current engine technology (less than 4 × 10^14^ particles per kg fuel).^12,13^

The volatile aerosol has a modal diameter of approximately 10 nm, which varies depending on the age of the exhaust plume and atmospheric conditions. The number of particles formed depends on the fuel sulfur content.^14^ Additionally, engine lubrication oil fumes are an important source for nonvolatile ultrafine particles.^15^ Recent studies indicate that lubrication oil fumes are efficient nucleation agents, a source for volatile aerosol that will not be addressed by replacing traditional jet fuels with sustainable aviation fuels.^16^ A key difference between sulfur acid-based volatile particles and aircraft soot particles is that volatile particles can increase in size through the condensation of gaseous compounds during the early stages of the exhaust plume,^17^ whereas aviation soot particles remain almost at the same size. Soot particles can also serve as condensation nuclei for organic species and sulfuric acid and therefore affect the nucleation and growth of volatile particles.^13^ Research aircraft studies conducted at cruising altitude have revealed that the growth of volatile particles continues within the jet regime for up to 10 s after the plume is released.^14^

During the early stages of the aircraft exhaust plume, volatile and nonvolatile particles interact through coagulation, which can result in a sulfuric acid coating on the hydrophobic aviation soot particles. Detailed studies on aircraft soot have demonstrated a significant increase in the ability of soot particles to act as cloud condensation nuclei as a result of this coating.^18^ While contrail formation requires the activation of aviation soot particles for droplet formation and subsequent freezing, their role in the formation of anthropogenically perturbed cirrus clouds is not understood. However, aggregated aviation soot particles have been found in contrail ice crystals at a higher concentration than in natural cirrus.^19^

All available studies on the aerosol particle properties within the aircraft exhaust plume are based on measurements in engine testing facilities at ground level,^20,21^ at airports^22−24^ or on a few specialized research aircraft missions at cruise altitude.^14,13^ All of these types of measurements are important for the analysis of aircraft exhaust aerosol chemical and physical properties and have their individual advantages and disadvantages. When measuring the exhaust aerosol at ground testing facilities or at airports, the aircraft engines are running under very different environmental conditions (e.g., air pressure and temperature) compared to cruising altitude. Due to the complexity of missions where a research aircraft is flying in the exhaust plume directly behind another aircraft, the number of measurements and tested engine types is limited. Furthermore, in both cases, only the very fresh aerosol particles with an age of a few seconds are observed. It is yet to confirm that the aerosol properties used in the inventories for global climate models, which are based on this kind of measurement, also represent the further aged aerosol particles in the dispersion phase of the aircraft exhaust plume (starting 2 min after emission)^25^ at a scale that can be resolved by global models.

This study utilizes the IAGOS-CARIBIC data set (In-service Aircraft for a Global Observing System–Civil Aircraft for the Regular Investigation of the Atmosphere Based on an Instrument Container), which contains the aerosol microphysical measurements of randomly probed aircraft exhaust plumes that were crossed during 36 long-distance passenger aircraft flights to destinations worldwide.

## 2 Methods and Instrumentation

### 2.1 IAGOS and the IAGOS-CARIBIC Flying Laboratory

The European Research Infrastructure IAGOS (www.iagos.org) provides a long-term observation platform for measuring the composition of the atmosphere within the major global flight corridors at altitudes up to 13 km. IAGOS uses the two complementary approaches, IAGOS-CORE and IAGOS-CARIBIC. IAGOS-CORE operates automated instruments on a fleet of long-haul aircraft for the continuous measurement of the essential climate variables ozone, carbon monoxide, nitrogen oxides, carbon dioxide, methane, water vapor, aerosol, and clouds. All measurements discussed in this work were performed utilizing the IAGOS-CARIBIC flying laboratory as a platform, which deploys a comprehensive set of complex scientific instruments aboard an Airbus A340-600 operated by Deutsche Lufthansa. The IAGOS-CARIBIC flying laboratory is a specialized air-freight container with automated instrumentation for simultaneous measurements of atmospheric aerosol particles and gaseous compounds. The container was deployed monthly, typically for four consecutive long-distance flights. A more detailed description of the IAGOS-CARIBIC flying laboratory can be found in Brenninkmeijer et al.'s study.^26^ Here, the measurements of the aerosol particle microphysical properties and the total reactive nitrogen (NO_*y*_) mixing ratio for the period from July 2018 to March 2020 are used.

### 2.2 IAGOS-CARIBIC Aerosol Microphysical Instrumentation

The aerosol particle microphysical properties for the IAGOS-CARIBIC data set were measured using the IAGOS-CORE aerosol instrument. This instrument contains an optical particle counter (OPC; Grimm) and two butanol condensation particle counters (CPC, Grimm); for a detailed description and characterization, see Bundke et al.'s study.^27^ The OPC is providing the particle number concentration for the accumulation mode particles with diameters of *D* > 250 nm. The OPC accuracy is estimated to be 4 ± 1% by comparing extinction measurements of a CAPS (Cavity Attenuated Phase Shift) PM_ex_ instrument with calculated values from Mie theory using the OPC size distribution. The precision of the total particle number concentration is reported with an average instrument-to-instrument comparison of ≤1%. The two CPCs were operated with a particle diameter detection range of 15 nm to 3 μm. While one CPC was measuring the total particle number concentration, the other one was detecting the nonvolatile aerosol particles after being heated up to 250 °C in a thermal denuder. The thermal denuder consists of a heated stainless-steel tube with an inner diameter of 9 mm and a length of 0.2 m. The details of the thermal denuder heating characteristic are discussed by Bundke et al.^27^ Both CPC instruments report total particle number concentration with an accuracy of 3 ± 1.5% against a Faraday cup electrometer. Side-by-side operation of GRIMM CPC leads to an estimated precision of 3.4%. All parameters were measured with a 1 Hz temporal resolution. All instrument characterization measurements were conducted in the laboratory, simulating the in-air temperature and pressure conditions as well as possible. Additionally, these instruments and the IAGOS-CARIBIC container as a platform were constructed to work under conditions within the aircraft. Nevertheless, extreme events during flight may result in larger uncertainties.

### 2.3 IAGOS-CARIBIC NO_*y*_ Instrumentation

The NO_*y*_ mixing ratio for IAGOS-CARIBIC was measured with the chemiluminescence instrument from the German Aerospace Agency (DLR); see Stratmann et al.'s study^28^ for details. The statistical detection limits of this instrument for the 1 Hz data are 7 pptv for the NO measurements and 8 pptv for the NO_*y*_ measurements. Meanwhile, the overall uncertainty for the NO and NO_*y*_ measurements was reported by Stratmann et al.^28^ to be 8% (6.5%) for a volume mixing ratio of 0.5 ppbv (1 ppbv). Even though laboratory instrument characterization was accounting for normal conditions during operation on aircraft, some extreme events during flight may result in larger uncertainties.

### 2.4 Plume Detection and Analysis

**Figure 1:**
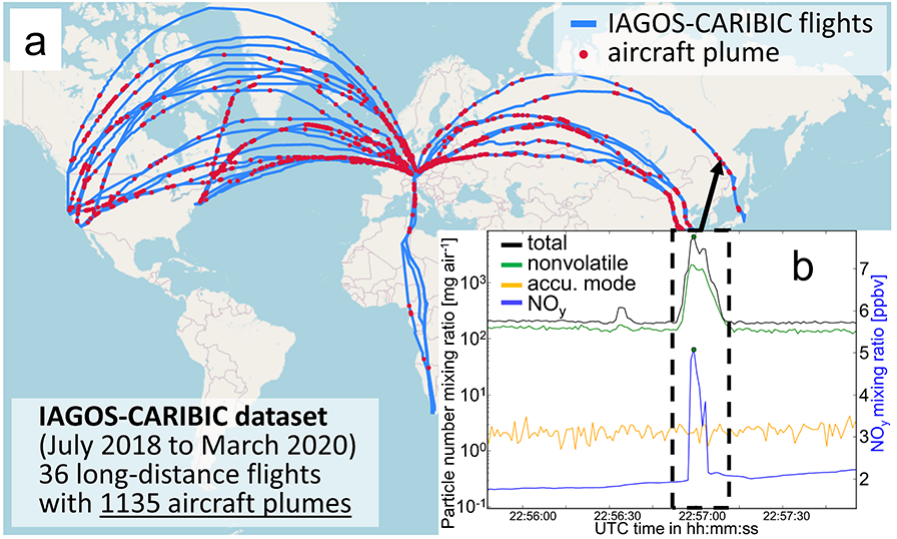
(a) World map with the IAGOS-CARIBIC flight tracks (blue lines) and the detected aircraft plumes (red dots). (b) Time series of the total (black line; *D* > 15 nm), nonvolatile (green line; *D* > 15 nm), and accumulation mode (orange line; *D* > 250 nm) particle number mixing ratio and the NO_*y*_ mixing ratio (blue line), exemplary for one aircraft plume. Map data © 2023 were obtained by OpenStreetMap.

Aircraft plumes were probed randomly during the IAGOS-CARIBIC flights. For this reason, a plume detection algorithm was implemented to identify the plume data within the full data set. Because aerosol particles and NO_*y*_ are coemitted by aircraft engines (see Figure 1), the plume detection algorithm was set up to search for simultaneously measured peaks in the 1 Hz resolved total aerosol particle number mixing ratio data and the NO_*y*_ mixing ratio data; see Figure 1b. For the detection of the individual peaks, the algorithm utilizes the find_peaks function from the python package scipy (version 1.8.0).^29^ To ensure that the detection of peaks from large-scale vertical transport and convection was minimized, the prominence, distance, and width parameters for the find_peaks function were individually optimized for the aerosol and NO_*y*_ data. The threshold parameter “prominence” was set to 300 particles mg^–1^ air and 0.2 ppbv for the aerosol and NO_*y*_ data, respectively, while the distance parameter was set to 10 s for both species. The width parameter was set to be between 2 and 40 s for the aerosol data and between 2 and 80 s for the NO_*y*_ data. The aircraft plumes were then identified by matching the detected peaks in the aerosol and NO_*y*_ data sets. A match was identified where a peak from the aerosol data set and a peak from the NO_*y*_ data set intersected within the peaks’ widths at half height. In the next step, all overlapping plumes were removed from the data set to avoid artifacts from mixed plumes of potentially different ages during the subsequent plume analysis.

For all the remaining “unambiguous” aircraft plumes, a plume analysis was executed to determine each plume’s excess of the NO_*y*_ mixing ratio and the aerosol particle number concentration of the total (*D* > 15 nm), nonvolatile (*D* > 15 nm), and accumulation mode aerosols (*D* > 250 nm), which were contributed by the aircraft to the background atmosphere. The background was fitted within a window with five times the width of the respective peak using the baseline function of the peakutils Python package (version 1.3.3).^30^ Species by species, the excess for each individual plume was then calculated by subtracting the integrated background values from the integrated values over the full peak and dividing it by the integration time over the peak’s width.

### 2.5 Plume Dispersion Model

A plume dispersion model is applied to estimate the age of the observed plumes by using NO_*y*_ as a chemically inert tracer (<18 h)^31^ during plume evolution in the plume dispersion phase. First, the concentration of NO_*y*_ is calculated at the initial time step (*t*_0_), which corresponds to a specific initial plume area (*A*_0_ = 0.15 km^2^; calculated based on eq 6 in Petry et al.'s study^31^). This initial time step represents the end of the vortex regime and the beginning of the dispersion phase, about 2 min after the emission (see Kärcher et al.'s study^25^). As the next step, the dispersion of NO_*y*_ within the growing plume area as a function of time is modeled following a Gaussian theory.^32^ Since NO_*y*_ is emitted in the form of NO_*x*_, the initial concentration of NO_*y*_, denoted as *C*_0_, is calculated from the aircraft NO_*x*_ emission rate using eq 1 and is used to initialize the plume model using parameter values from Lee et al.^33^ and Petry et al.^31^
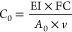
1

In this equation, *C*_0_ represents the concentration of NO_*y*_ in g m^–3^ at time *t*_0_, EI is the emission index of NO_*x*_ (which is assumed to be equivalent to NO_*y*_ at this plume age) in g kg^–1^, FC is the fuel consumption rate in kg s^–1^, *A*_0_ is the initial area in m^2^, and *v* is the aircraft speed in m s^–1^. For *v*, a typical aircraft speed of 870.7 km h^–1^ (about 240 m s^–1^) for a Boeing B-747 aircraft^31^ was used. The dilution of emitted species within the aircraft exhaust plume is dependent on the dispersion dynamics, which are influenced by parameters such as diffusion coefficients (vertical, horizontal, and shear) and wind shear.^31^ These parameters control the dispersion processes within the expanding plume. Although turbulence is not modeled explicitly, it is implicitly accounted for in the description of the diffusion process based on Petry et al.^31^ The evolution of the NO_*y*_ concentration during the dispersion phase is simulated by the plume model with 5 min time resolution over 24 h. For an aircraft speed of about 240 m s^–1^, one time step would correspond to a distance traveled by the aircraft of 72 km.

## 3 Results and Discussion

### 3.1 Plume Characteristics and Plume Age

**Figure 2:**
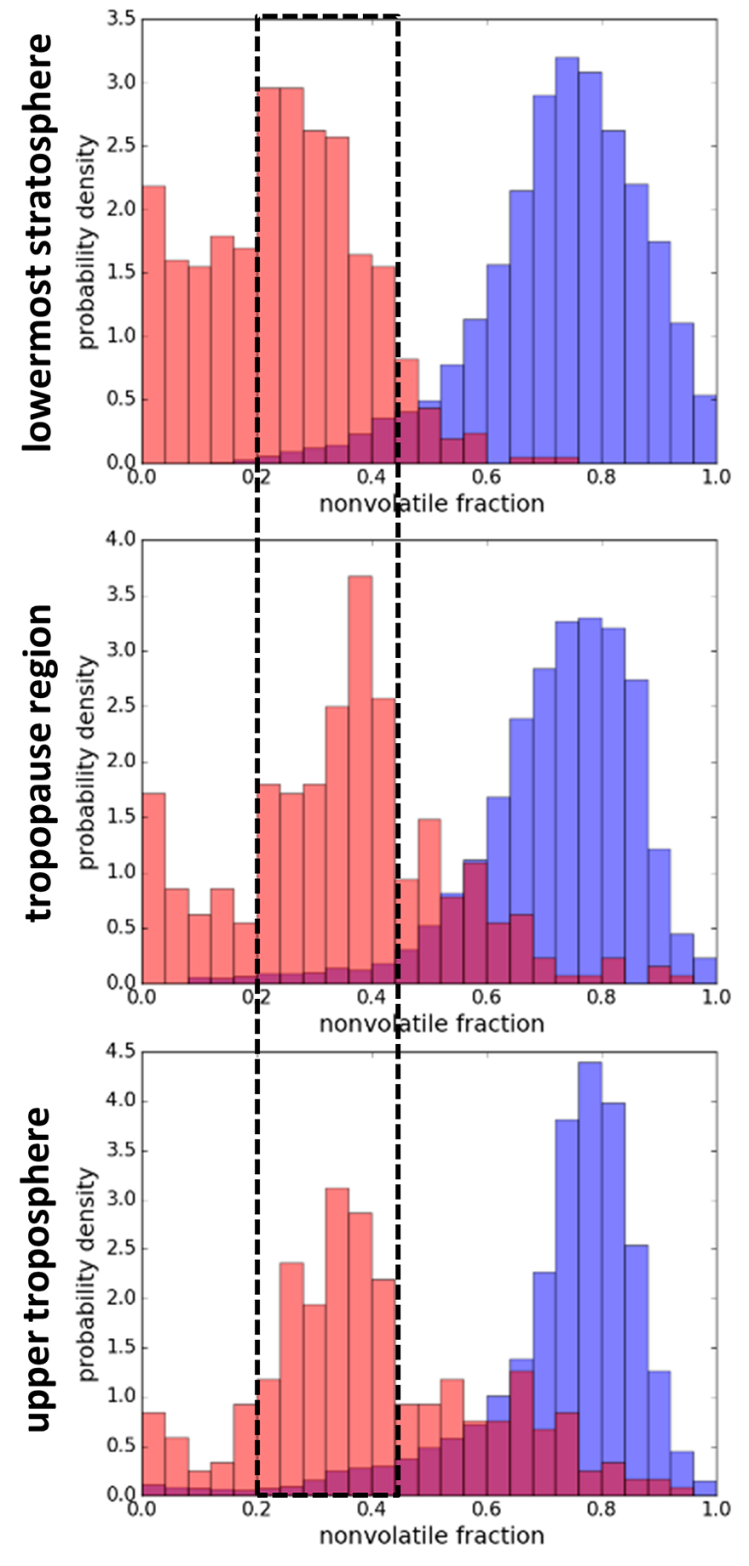
Nonvolatile fraction distributions (*D* > 15 nm) for the plume excess aerosol (red) and the background aerosol (blue) separated for the upper troposphere, tropopause region, and lowermost stratosphere. The black dashed box indicates the range of the main mode of the plume excess nonvolatile fraction.

For the period between July 2018 and March 2020, all measurements required for this analysis are available from a subset of 36 of the total 42 IAGOS-CARIBIC flights. Based on the NO_*y*_ and aerosol particle measurements, the previously described plume detection algorithm identified 1135 unique aircraft plumes. Figure 1 shows a world map with all detected unique aircraft plumes as red dots on the IAGOS-CARIBIC flight tracks (blue lines). These flights covered routes between Munich (Germany) and destinations in North America, South Africa, and East Asia, thereby providing a solid statistical basis and global insight into the impact of aviation on aerosol and trace gas properties within the main flight corridors. Utilizing a data set including nearly 2 years of atmospheric observations, the detected aircraft plumes are also covering all annual seasons. Of in total 1135 detected aircraft plumes, 200 were observed during winter (December to February), 48 in spring (March to May), 513 during summer (June to August), and 374 in autumn (September to November). It must be noticed that the number of detected plumes does not represent the actual occurrence frequency of aircraft plumes in the atmosphere during the different seasons but is primarily driven by the number of IAGOS-CARIBIC flights on different flight routes during those seasons. For further analysis, all detected exhaust plumes were categorized in terms of the upper troposphere (pressure level relative to the thermal tropopause: Δ*p*_ttp_ > 15 hPa), tropopause region (Δ*p*_ttp_ ± 15 hPa), and lowermost stratosphere (Δ*p*_ttp_ < −15 hPa) using the ECMWF (European Centre for Medium-Range Weather Forecasts)^34^ reanalysis thermal tropopause data. This results in 24% upper tropospheric plumes, 28% plumes in the tropopause region, and 48% of the plumes observed in the lowermost stratosphere. Further analysis shows that the main mode of the nonvolatile particle fraction (for particles with *D* > 15 nm) inside the plume is between 0.2 and 0.4 for all those atmospheric layers (see Figure 2), while this fraction is about 0.8 for the background aerosol. Weinzierl et al.^35^ explain the link between the volatile fraction and the mixing state of the aerosol based on the change in size distribution for internally and externally mixed aerosols. This finding indicates that the plume aerosol is more externally mixed, while the background aerosol at all atmospheric layers is mostly internally mixed. The observed independence of the plume aerosol properties from the background aerosol allows for plume aerosol parametrization independent of the flight altitude and respective atmospheric layers within the main flight corridors.

**Figure 3:**
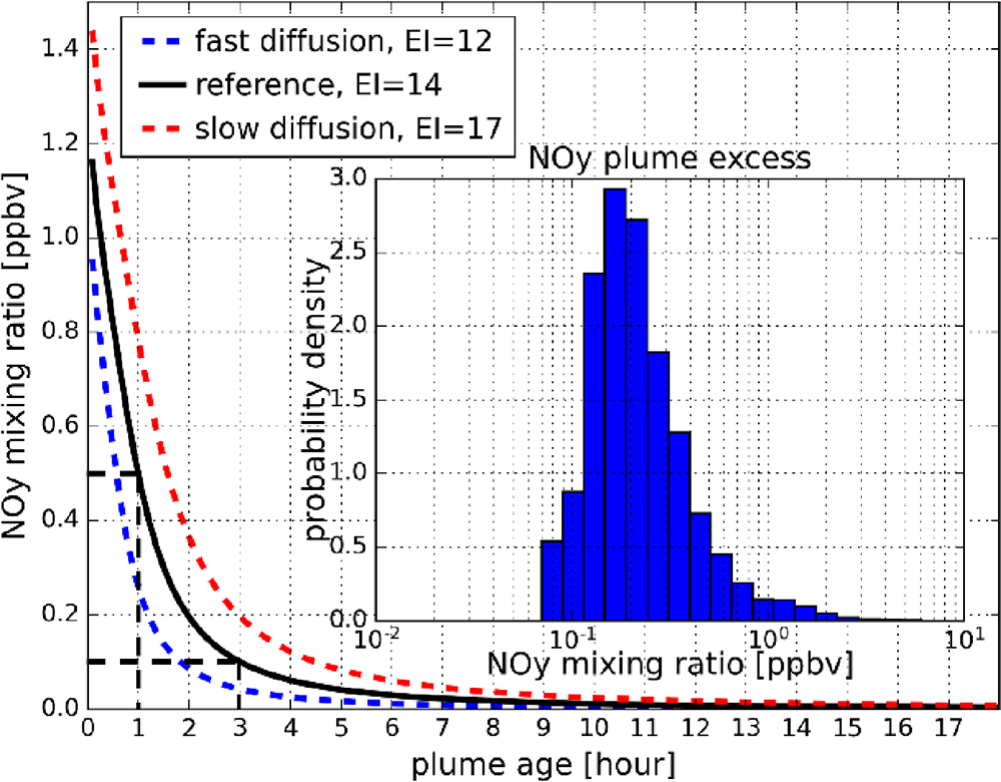
Simulated NO_*y*_ mixing ratio as a function of the plume age resulting from plume dispersion modeling. With the reference case as a black line and the histogram of the observed NO_*y*_ plume excess mixing ratio, the black dashed lines indicate the mean plume age of 1 to 3 h resulting from the main mode of the NO_*y*_ mixing ratio distribution between about 0.1 and 0.5 ppbv.

In contrast to aircraft exhaust observations at ground testing stations and airborne measurements where the exhaust plume is probed directly behind another aircraft, here, the plumes were observed randomly at very different ages. The previously described plume dispersion model was used to estimate the age range of the observed aircraft plumes using the NO_*y*_ mixing ratio as a proxy. Figure 3 depicts the modeled NO_*y*_ mixing ratio of an aircraft plume in the dispersion phase as a function of the plume age. The black line represents the reference case with a NO_*y*_ emission index of 14 g kg^–1^. Additionally, an upper limit case (red dotted line) with slower diffusion and a NO_*y*_ EI of 17 g kg^–1^ and a lower limit case for the plume age (blue dotted line) with faster diffusion and a NO_*y*_ EI of 12 g kg^–1^ were calculated. For the short plume ages discussed here, the reference case and range of the NO_*y*_ emission indices used were set to be equal to the fleet average NO_*x*_ EIs from 2010 reported by Lee et al.^33^ The resulting range in plume age for a constant NO_*y*_ mixing ratio shows clearly that this method can only be used to estimate the age range of all observed aircraft plumes statistically and not the precise age of every single plume individually.

The histogram of the NO_*y*_ plume excess mixing ratio (see Figure 3) has its main mode between 0.1 and about 0.5 ppbv. Comparing this range of NO_*y*_ mixing ratios with the results from the plume dispersion model translates into a plume age range for the observed aircraft plumes of about 1 to 3 h. In contrast to the only few second-old aerosol particles observed directly behind aircraft engines, the plume excess aerosol measured by IAGOS-CARIBIC can be considered as aged aerosol, where the aerosol particle number concentration and nonvolatile fraction are mainly driven by dispersion and processes like nucleation and coagulation are not efficient anymore.

### 3.2 Accumulation Mode Particles

One important factor for the climate impact of aviation is the presence of large accumulation mode particles, which by themselves have a stronger radiative impact compared to smaller aerosol particles but can also act as efficient condensation nuclei and therefore enhance the formation of contrail cirrus clouds.^36^ The analysis of all detected aircraft plumes showed no detectable and statistically significant enhancement of the accumulation mode particle number (*D* > 250 nm) compared to the background variability. The time series of the aerosol particle number mixing ratios (total aerosol as the black line, nonvolatile aerosol as the green line, and accumulation mode aerosol as the orange line) and the NO_*y*_ mixing ratio (blue line) of one example plume is shown in Figure 1b. This example case visualizes that no enhancement from the background variability of particles with diameters >250 nm can be observed, even though this is one of the youngest and therefore least diluted plumes observed, with a NO_*y*_ mixing ratio peaking at about 5 ppbv.

### 3.3 Particle Number Emission Index Calculation

**Figure 4:**
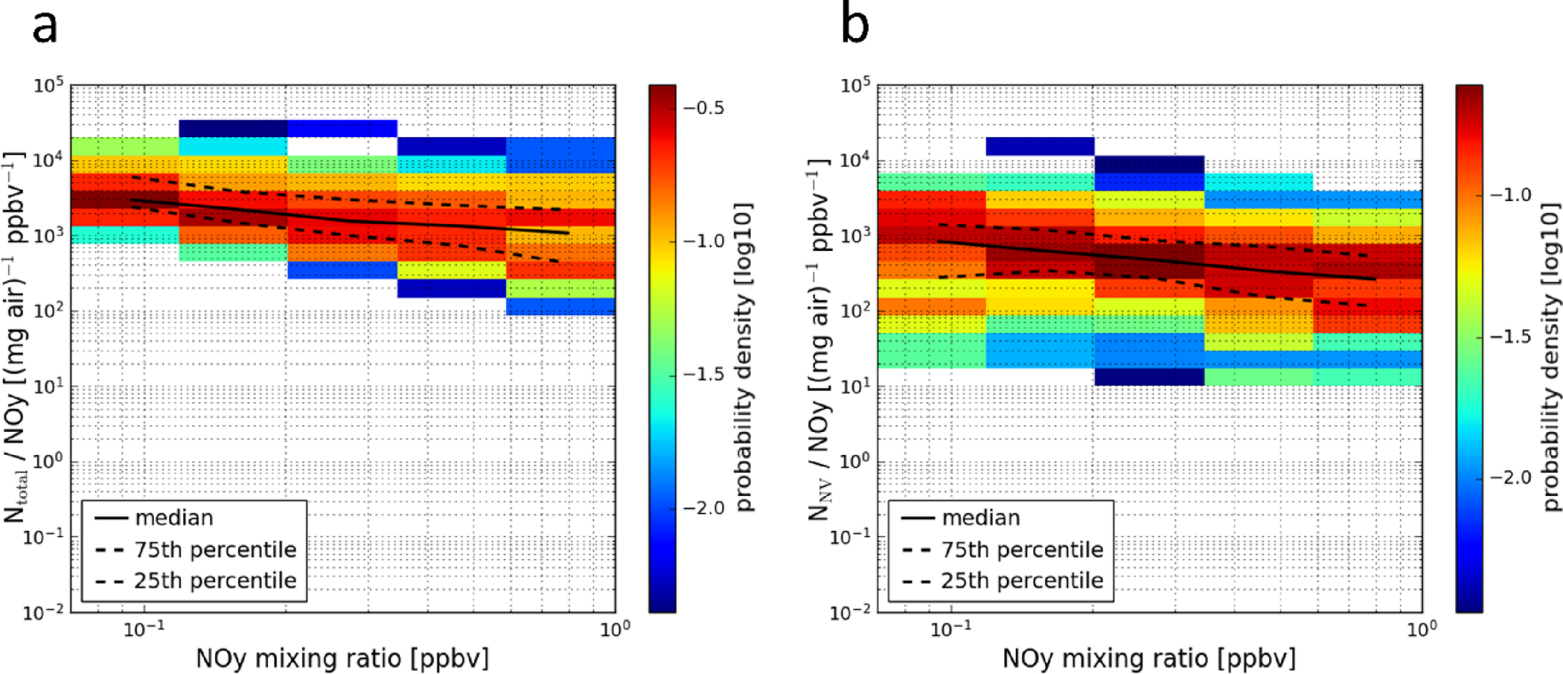
Heatmaps of the plume individual total (a) and nonvolatile (b) particle number mixing ratios normalized by their corresponding NO_*y*_ mixing ratios as a function of the NO_*y*_ mixing ratios. The median and the 25th and 75th percentiles for each NO_*y*_ bin are indicated by a black line and dotted black lines, respectively. The probability density within each NO_*y*_ mixing ratio bin is color-coded.

Global climate models are driven by emission data provided in global emission inventories. These, however, only provide information about the emitted mass of various compounds, such as soot and NO_*x*_, but do not include particle number emissions. These need to be derived from the mass emissions by assuming typical size distributions of the emitted particles. These assumptions are usually based on measurements and implicitly account for particle transformation processes occurring during the plume dispersion phase that cannot be resolved by the models due to their coarse resolution.^5,7^ For this reason, only the data of plumes that are in a state where the change in the aerosol number is mostly driven by the plume dispersion should be used. This way, one can make sure that the calculated particle number emissions represent the exhaust aerosol in a processing state that can be resolved with the large-scale model grid boxes (∼100 km) by processes typically represented by climate models. In Figure 4, heatmaps of the plume total (a) and nonvolatile (b) particle number mixing ratios normalized by their corresponding NO_*y*_ mixing ratios are shown as a function of the NO_*y*_ mixing ratios. The black line and black dotted lines represent the median and the 25th and 75th percentiles for each NO_*y*_ bin. Assuming that NO_*y*_ is an inert tracer within the timescales of the detected plumes’ age, the particle number mixing ratio normalized by the NO_*y*_ mixing ratio should be constant as a function of the NO_*y*_ mixing ratio when the change in particle number is only driven by the plume dispersion. The deviation from this constant behavior (visible in Figure 4) may be explained by the limitations of the sampling and calculation of the plumes’ particle and NO_*y*_ excess, especially for older plumes with very low excess mixing ratios. As the plumes were encountered randomly at largely unknown plume positions, this may have caused uncertainties by differences in the response times of the NO_*y*_ and aerosol instruments. This uncertainty is minimized by analyzing statistically a large number of plumes. Considering these limitations, only a subset of the plume data with excess NO_*y*_ mixing ratios between 0.2 and 0.4 ppbv was used for the particle EI calculations.

**Figure 5:**
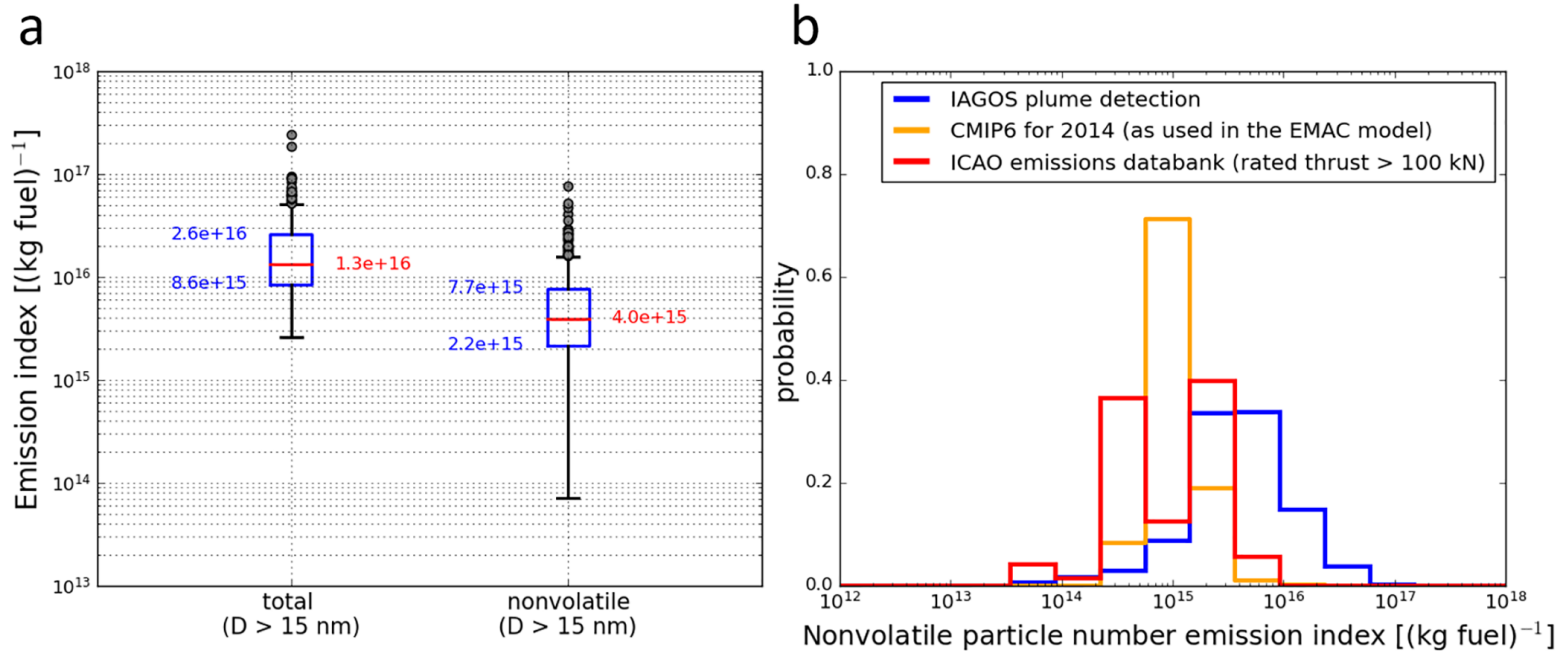
(a) Emission index of the total aerosol particles (left) and the nonvolatile aerosol particles (right) shown as a boxplot for all observed aircraft plumes within the IAGOS-CARIBIC data set. (b) Histogram of the nonvolatile particle number emission index calculated based on the IAGOS-CARIBIC data set (blue), derived from the CMIP6 inventory and used in the EMAC global model (orange), and reported in the ICAO emission databank for engines with thrust ratings greater than 100 kN (red).

For each aircraft plume that fulfilled this criterion, the total and nonvolatile particle EIs were calculated. The EIs were calculated by the method demonstrated by Voigt et al.^37^ who used the CO_2_ EI to calculate the particle EI, here using the mean aviation NO_*x*_ EI of 14 g (kg fuel)^−1^.^33^ This results in median particle number EIs of 1.3 × 10^16^ (kg fuel)^−1^ for the total aerosol (*D* > 15 nm) and 4 × 10^15^ (kg fuel)^−1^ for the nonvolatile aerosol (*D* > 15 nm). In Figure 5a, in addition to the median values (red line), the 1st and 99th percentiles (black vertical bar), 25th and 75th percentiles (blue box), and the outliers (gray dots) are presented as a boxplot.

### 3.4 Discussion of Resulting EIs with the Literature, Emission Databanks, and Model Emission Inventories

Volatile particle EIs normalized to a plume age of 3 s were reported by Schumann et al.^38^ to range between about 1 × 10^16^ and 2 × 10^17^ (kg fuel)^−1^ for fuel sulfur contents between 2 and 3000 μg g^–1^. For 2006, the average fuel sulfur content was estimated to be between 550 and 750 μg g^–1^, well below the specification limit of 3000 μg g^–1^.^39^ According to Schumann et al.,^38^ this would result in volatile particle EIs well below 1 × 10^17^ (kg fuel)^−1^. Together with the lower CPC cutoff diameter of 3 nm used by Schumann et al.^38^ (15 nm for the IAGOS-CARIBIC data set), the difference to the median EI for volatile particles of about 1 × 10^16^ (kg fuel)^−1^ for the IAGOS-CARIBIC observations can be explained by further processing (e.g., coagulation with other volatile and nonvolatile particles) of the only 3 s-old plume aerosol.

The nonvolatile plume excess particles with diameters larger than 15 nm can be assumed to consist mainly of soot. Directly emitted metal particles from engine erosion and the combustion of fuel containing trace metal impurities or metal particles that enter the exhaust with the fuel only have EIs of 10^7^ to 10^8^ (kg fuel)^−1^.^40^ Anderson et al.^41^ reported nonvolatile particle EIs ranging from 0.5 × 10^15^ to 10 × 10^15^ (kg fuel)^−1^, while the EI values from Schumann et al.^38^ are between about 0.2 × 10^15^ and 1.8 × 10^15^ (kg fuel)^−1^. For two reference Jet A1 fuels, Voigt et al.^37^ measured nonvolatile particle EIs of 3.8 × 10^15^ and 4.9 × 10^15^ (kg fuel)^−1^ following an Airbus A320 aircraft. With a median EI resulting from the IAGOS-CARIBIC data set at 4 × 10^15^ (kg fuel)^−1^ and the 25th and 75th percentiles at 2.2 × 10^15^ and 7.7 × 10^15^ (kg fuel)^−1^, respectively, these observations are at the upper end of the EI range found by inflight measurements of very fresh exhaust plumes (age of a few seconds).

The International Civil Aviation Organization (ICAO) engine emission databank contains exhaust information like particle EI.^42^ The data are provided by the engine manufacturers, while the measurements at ground testing facilities must comply according to the procedures in ICAO Annex 16, Volume II. The first data on nonvolatile particle measurements included in the ICAO database are from the year 2013.^42^ A subset of engines with a thrust rating >100 kN was selected to represent the most common engine types used within the international main flight corridors. The histogram of the EI under approach condition, corrected for system losses from the ICAO database subset (see Figure 5b), shows one mode at smaller EI values and one within the main mode of the IAGOS-CARIBIC EI distribution. By selecting engine types with increasing thrust rating, the number of engine types with low EI values decreases. However, the histogram of the ICAO data cannot directly be compared to the IAGOS-CARIBIC observation because the ICAO database distribution only represents the number of engine types in the database and is not weighted by the number of the corresponding engines of each type that are currently used in the global fleet. But it still gives an indication that the globally observed EIs are at the upper end of the values reported by the engine manufacturers. Besides the different environmental conditions at ground testing, the engines used for emission measurements by the manufacturers can be expected to be either newly built or very well maintained. This would cause lower particle emissions compared to older engines of the same type.

To compare the nonvolatile particle number EI calculated based on the IAGOS-CARIBIC data set with the inventories currently used for climate models, the input data used in the simulations by Righi et al.^7,43^ with the global climate-chemistry model EMAC (ECHAM/MESSy Atmospheric Chemistry)^44^ were considered. This is based on the soot and CO_2_ emission data from the CMIP6 emission inventory for 2014,^45^ which allowed to derive the soot EI (assuming a mean CO_2_ EI of 3160 g kg^–1^).^33^ This was then converted to a particle number EI using the size distribution parameters given in Petzold et al.'s study^11^ which are based on in situ measurements. In Figure 5b, the resulting global EI values are depicted as a histogram together with the IAGOS-CARIBIC EI histogram. Even though the main mode of the CMIP6-based EI distribution has its peak at lower EI values as the main mode peak of the IAGOS-CARIBIC data, the distributions are widely overlapping. The median value of 1 × 10^15^ (kg fuel)^−1^ for the CMIP6-based EI is a factor of 4 lower than the 4 × 10^15^ (kg fuel)^−1^ for the nonvolatile particle EI from IAGOS-CARIBIC. For this comparison, several uncertainties must be considered. The measurement and data processing uncertainties for the IAGOS-CARIBIC data are assumed to have the smallest impact. The variability of the NO_*x*_ EI introduced by the global mean value of 14 g kg^–1^ and its range (12–17 g kg^–1^),^33^ together with the assumption of NO_*y*_ being an inert tracer and being equal to NO_*x*_ for the observed plume ages, is considered to be one of the main contributors. However, since we sampled randomly from a wide range of aircraft types, the application of the fleet average NO_*x*_ EI seems to be justified. Potential removal of aviation aerosol particles due to deposition on contrail ice particles and subsequent sedimentation could result in lower particle EIs for plumes affected by contrail formation. The largest uncertainty is the sampling of the plumes at random positions. The impact of the uncertainties introduced by the two latter processes is minimized statistically by analyzing a large set of plumes. For the model, the EI calculation based on CMIP6 relies on the CO_2_ and soot emission inventory data as well as the mean CO_2_ EI.

Considering these uncertainties, on the one hand, this comparison reveals that the soot number EIs used in the models are within the range of the EIs resulting from the IAGOS-CARIBIC data set, representing a significant part of the global fleet within the main flight corridors. On the other hand, the identified discrepancy could indicate a potential underestimation of the number concentration of aviation-induced soot particles in the models, which should be considered when interpreting modeled aviation soot climate effects as described, for instance, by Righi et al.^7^ Using the IAGOS-CARIBIC data set with its unique advantages of in situ observations with the global coverage of the main flight corridors over a period of nearly 2 years and statistics over about 1100 aircraft plumes underlines the representatives of the measured particle EIs (EI_total_ = 1.3 × 10^16^ (kg fuel)^−1^ and EI_nonvolatile_ = 4 × 10^15^ (kg fuel)^−1^) and their respective ranges for the emissions of the mean global fleet. Furthermore, no enhancement above the background variability of large accumulation mode particles (*D* > 250 nm) could be detected for the aircraft plume excess. The fact that after 1–3 h, the main mode of the plume aerosol nonvolatile fraction distribution is still between 0.2 and 0.4 indicates that plume internal aerosol processing has come to a steady state and that the aviation exhaust aerosol almost remains in its emission state during plume expansion.

## Abbreviations

*A*_0_: initial plume area
BC: black carbon
*C*_0_: initial concentration
CARIBIC: Civil Aircraft for the Regular Investigation of the Atmosphere Based on an Instrument Container
CO_2_: carbon dioxide
CPC: condensation particle counter
*D*: diameter
DLR: Deutsches Zentrum für Luft- und Raumfahrt (English: German Aerospace Agency)
Δ*p*_ttp_: pressure relative to thermal tropopause
ECMWF: European Centre for Medium-Range Weather Forecasts
EI: emission index
EMAC: ECHAM/MESSy Atmospheric Chemistry
FC: fuel consumption rate
HC: hydrocarbon
IAGOS: In-service Aircraft for a Global Observing System
NO_*x*_: nitrogen oxides
NO_*y*_: total reactive nitrogen
OC: organic carbon
OPC: optical particle counter
ppbv: parts per billion by volume
*t*_0_: initial time step
*v*: aircraft speed
